# Off-stoichiometry and molybdenum substitution effects on elastic moduli of B1-type titanium carbide

**DOI:** 10.1038/s41598-023-40969-x

**Published:** 2023-08-21

**Authors:** Shuntaro Ida, Kotaro Hoshizaki, Takahiro Kaneko, Xi Nan, Nobuaki Sekido, Kyosuke Yoshimi

**Affiliations:** Department of Materials Science, Graduate School of Engineering, Tohoku University, 6-6-02 Aramaki Aza Aoba, Aoba-ku, Sendai, 980-8579 Japan

**Keywords:** Materials science, Structural materials, Ceramics, Metals and alloys

## Abstract

B1-type MX ceramics are composed of transition metals (M) and C, N, and/or O (X) occupying the M and X sites, respectively, and having M**–**X nearest neighbor (NN) bonds and M**–**M and X**–**X next nearest neighbor (NNN) bonds. Substitution of the elements and the formation of structural vacancies in B1-type ceramics change the numbers and strengths of the bonds, leading to novel properties. The change in elastic modulus of off-stoichiometric TiC in equilibrium with a Ti**–**Mo solid solution phase was experimentally investigated based on the rule of mixtures from the Voigt model. The experimentally obtained values agreed well with the results of density functional theory calculations. The bulk modulus (*K*) of TiC increased from 205.6 to 239.2 GPa as the fraction of Ti sites occupied by Mo increased from 0.11 to 0.33, whereas the Young’s modulus (*E*) and the shear modulus (*G*) remained nearly constant. On the other hand, all three elastic moduli decreased with increasing vacancy fraction at the C sites. These results suggest that the M–X bond strength should be the dominant factor in these moduli and the effect of M–M bond on* K* is greater than that of *G* and *E*.

## Introduction

B1-type MX compounds composed of transition metals (M) and C, N, and/or O (X), occupying the M and X sites, respectively, mainly with covalent M–X bonds, exhibit attractive material properties, such as low density, high melting point, high hardness, good wear resistance, and moderate electric conductivity^1–3^. Consequently, these ceramic phases are widely used in thin films, as coatings for cutting tools, as hard phases in cermets, etc., and they are also found as nanosized precipitates in some steels^2,4–8^. One drawback of B1-type compounds is their brittleness; for example, the fracture toughness of stoichiometric TiC is only about 3 MPa(m)^1/2^^9,10^. If this poor toughness could be improved without a commensurate decrease in strength, the applications of the resulting ceramics would further expand as ultra-high temperature materials that can be used to improve the energy efficiency of gas turbines and jet engines and for thermal-protection systems in spacecraft bodies^11–15^.

B1-type MX compounds can have relatively high degrees of off-stoichiometry compared with other ceramics, such as SiC and MAX phases^13–17^. For example, the compositional region for Ti_2_AlC is very narrow in the Ti–Al–C ternary system, even at 1300 °C^18^. On the other hand, the TiC phase region in the Mo–Ti–C ternary system expands toward both the Ti-rich and the Mo-rich regions, and these off-stoichiometries change the properties of the material by changing the types and numbers of bonds due to elemental substitution and the formation of structural vacancies^19^.

In the field of intermetallic compounds, there is a long history of studies on how material properties change due to off-stoichiometry. For example, B2-type intermetallic compounds have been extensively studied for the effects of off-stoichiometry on their defect structures and properties^20–27^. Here, it is well known that the defect structure of B1-type MX compounds is of the vacancy type in the transition-metal-rich region^28–31^. In the early studies, shifts in the binding energy and band structure of off-stoichiometric TiC were already discussed^32,33^. Over the last two decades, the phase stabilities and elastic moduli of B1-type MX compounds relating to the defect structure have been investigated by means of density functional theory (DFT) calculations^34–37^. Elastic properties of multicomponent B1-type MX compounds with vacancies^38,39^ or without vacancies^40–43^ have also been investigated by means of DFT calculations. It is more meaningful to investigate further the off-stoichiometric effect with structural vacancies on the material properties of multicomponent B1-type MX compounds experimentally and computationally.

The off-stoichiometry may slightly improve the toughness and/or plastic deformability of B1-type MX compounds. Fe–Ti–C alloys containing near-stoichiometric TiC in equilibrium with an Fe phase exhibited low ductility, and their elongation simply decreased with increasing the volume fraction of TiC^44^. On the other hand, Ti–Mo–Al alloys containing off-stoichiometric TiC exhibited better deformability than the alloys lacking TiC^45^. TiC-added Mo–Si–B (MoSiBTiC) alloys exhibit greater strength at high temperatures and better fracture toughness at room temperature than Mo–Si–B alloys^46–49^. TiC in MoSiBTiC alloys is in equilibrium with Mo solid-solution and Mo_5_SiB_2_ phases, and it contains more than 20 at% of Mo and less than 50 at% of C^50^. Off-stoichiometric TiC in the fractography of the MoSiBTiC alloys showed river patterns, suggesting a small amount of plastic deformation. Moreover, the fracture toughness of MoSiBTiC alloys increased with increasing total volume fractions of Mo and TiC phases^48^. These results suggest that the off-stoichiometric TiC deformed plastically and acts as a fracture resistant phase. On the other hand, Sangiovanni et al. reported that high-entropy refractory ceramics with B1-type structures exhibit plasticity using both ab initio molecular dynamics simulations and nanoindentation^51^. These results suggest that TiC might acquire slight plastic deformability through off-stoichiometry, and the plastic behavior should be related to the elastic properties of the alloys again.

Therefore, the elastic modulus change by off-stoichiometry in (Ti, Mo)C_*x*_ in equilibrium with a Mo–Ti solid solution is experimentally investigated in this study. DFT calculations are also used to estimate the elastic properties of the off-stoichiometric (Ti, Mo)C_*x*_ in the Mo–Ti–C ternary system. By comparing the elastic moduli obtained from the experiments with those from the DFT calculations, factors controlling the elastic properties of the off-stoichiometric B1-type (Ti, Mo)C_*x*_ are clarified, and the material property change by off-stoichiometry in the multicomponent (Ti, Mo)C_*x*_ is discussed.

## Results

**Figure 1:**
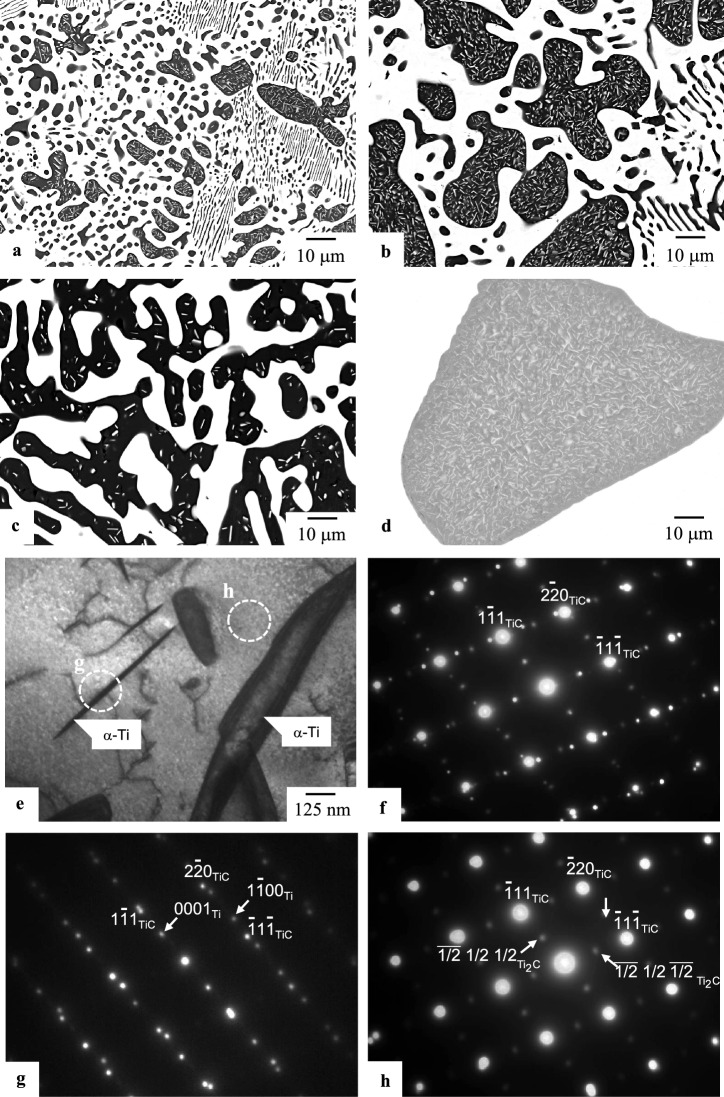
Backscattered electron images (BEIs) and TEM images of the microstructures of alloys in the ternary system after heat treatment at 1800 °C for 72 h (**a**–**c**) and in the binary system after heat treatment at 1500 °C for 72 h (**d**–**h**): (**a**) BEI of Mo–20.0Ti–20.0C, (**b**) BEI of Mo–37.9Ti–25.0C, (**c**) BEI of Mo–53.2Ti–25.0C and (**d**) BEI of Ti–10.0C, (**e**) Bright-field image (*B* = 110_TiC_) of Ti–5C, (**f**) Selected-area diffraction pattern (SADP) taken from whole area of (**e**), (**g**,**h**) SADP taken from the areas shown by the corresponding dotted circles in (**e**).

The microstructures of the alloys studied are shown in Fig. 1. Alloys in the ternary system consisted of a Mo phase (A2-type structure) with a bright contrast and a TiC phase with a dark contrast in the SEM-backscattered electron images (BEI) (Fig. 1a–c). Part of the Mo phase precipitated in TiC. Alloys in the Ti–C binary system were composed of an α-Ti phase (A3-type structure) with a bright contrast and TiC with a dark contrast in the BEI (Fig. 1d). The bright phase also precipitated in TiC. Furthermore, a finer phase with a sharper interface than the α-Ti phase shown in Fig. 1d was also observed in the TEM-bright field image (TEM-BFI; Fig. 1e). The finer phase was identified as an α-Ti phase, and the finer α-Ti phase had a habit plane of (111)_TiC_ (Fig. 1f,g). The coarse α-Ti phase shown in Fig. 1d with a bright contrast is likely to be formed by the transformation of the β-Ti phase (A2-type structure) formed during heat treatment at 1500 °C, whereas the finer α-Ti phase shown in Fig. 1e would precipitate during cooling after heat treatment. Superlattice spots of the vacancy-ordered Ti_2_C phase, as mentioned in some reports in the literature^52–55^, were also observed in the TiC matrix (Fig. 1f,h). Two types of structure of the Ti_2_C phase have been reported: an *R*$\stackrel{\mathrm{-}}{3}$*m* type and an *Fd*3*m* type. However, it was found that a higher spatial resolution would have been required to identify the structure of the Ti_2_C phase.

**Figure 2:**
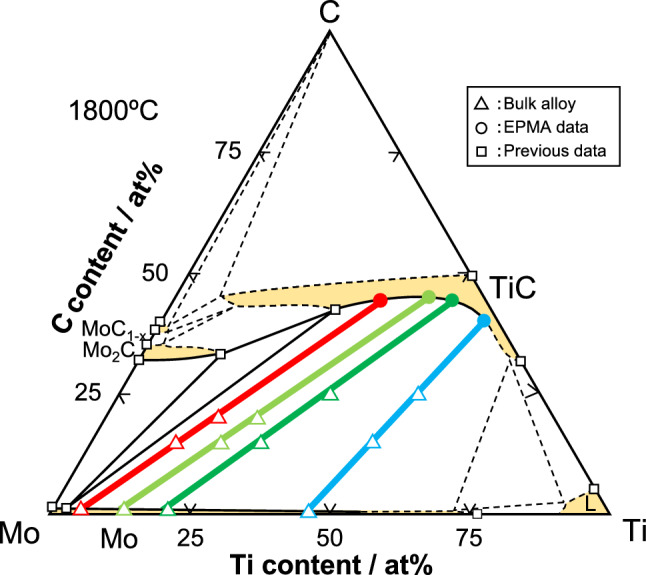
Isothermal section at 1800 °C of the Mo–Ti–C ternary system^19,56,57^. The tie lines of each alloy in the Mo/TiC two-phase region of Mo–Ti–C ternary system are shown by the colored lines.

**Figure 3:**
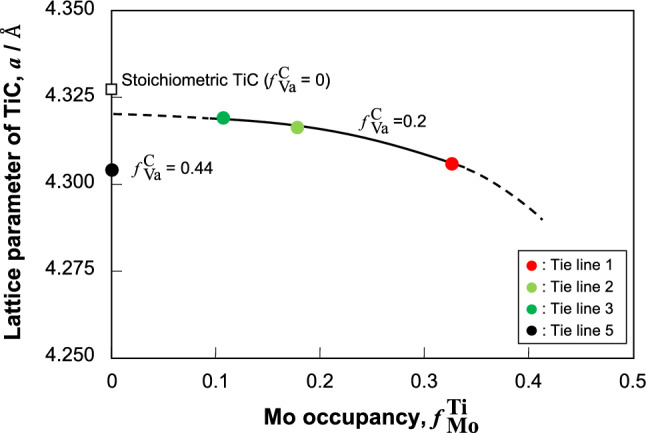
Changes in the lattice parameter of TiC with the terminal compositions of Tie Lines 1–3 ($f{}_{\text{Va}}^{\text{C}}$ = 0.2) as a function of the Mo fraction, together with binary TiC data ($f{}_{\text{Va}}^{\text{C}}$ = 0)^3^.

An isothermal section of the Mo–Ti–C ternary system at 1800 °C is shown in Fig. 2^19,56,57^. All compositions of the Mo–Ti–C ternary alloys examined in this study are plotted on the colored tie lines drawn in the Mo/TiC two-phase region. Hereafter, these tie lines are referred to as Tie Lines 1–4 from the Mo–rich composition, respectively, and the tie line in the Ti/TiC two-phase region in the Ti–C binary system is referred to as Tie Line 5. The terminal compositions of TiC for Tie Lines 1–3 had almost the same C content, whereas the C content markedly decreased in Tie Lines 4 and 5. The constituent phases, the composition and lattice parameter of the constituent phases, and the volume fraction of TiC are summarized in Table 1. All TiC phases measured in equilibrium with the solid-solution phase had a C-poor composition. The lattice parameters of TiC measured in this study were smaller than that of stoichiometric TiC (4.327 Å)^3^. This can be attributed to a vacancy defect structure at the C site, as previously reported^28–31^. Therefore, the structural defect in off-stoichiometric TiC in the Mo–Ti–C ternary system can be assumed to be a substitution of Mo at Ti sites and the formation of vacancies at the C sites. The Mo-fraction dependence at the Ti sites and the vacancy-fraction dependence of TiC are divided by the Mo fraction at Ti sites ($f{}_{\text{Mo}}^{ \, {\text{Ti}}}$) and the vacancy fraction at C sites ($f{}_{\text{Va}}^{ \, {\text{C}}}$). These are given by the following equations:

1 $$f{}_{\text{Mo}}^{\text{Ti}}\text{ = }x{}_{\text{Mo}}^{\text{TiC}}\text{ / (}x{}_{\text{Mo}}^{\text{TiC}}\text{ + }x{}_{\text{Ti}}^{\text{TiC}}\text{)},$$

2 $$f{ }_{{{\text{Va}}}}^{{\text{C}}} {\text{ = 1 - }}x_{{\text{C}}}^{{{\text{TiC}}}} { / (}x_{{{\text{Mo}}}}^{{{\text{TiC}}}} { + }x_{{{\text{Ti}}}}^{{{\text{TiC}}}} {)},$$

where $x{}_{\text{Mo}}^{\text{TiC}}$, $x{}_{\text{Ti}}^{\text{TiC}}$, and $x{}_{\text{C}}^{\text{TiC}}$ are the Mo, Ti, and C compositions of TiC, respectively. Therefore, increases in $f{}_{\text{Mo}}^{ \, {\text{Ti}}}$ and $f{}_{\text{Va}}^{ \, {\text{C}}}$ imply a substitution of Mo at Ti sites and the formation of vacancies at C sites, respectively. The lattice parameters of TiC are summarized by using $f{}_{\text{Mo}}^{ \, {\text{Ti}}}$ and $f{}_{\text{Va}}^{ \, {\text{C}}}$ in Fig. 3. In the case of TiC with $f{}_{\text{Mo}}^{ \, {\text{Ti}}}$= 0, the lattice parameter of stoichiometric TiC was the highest, and the lattice parameter of TiC decreased with the formation of vacancies. The lattice parameters of TiC with almost the same values of $f{}_{\text{Va}}^{ \, {\text{C}}}$ decreased with increasing $f{}_{\text{Mo}}^{ \, {\text{Ti}}}$^3^. This change corresponds to a difference in atomic size, as the Mo atom is smaller than the Ti atom^58^. Therefore, it can be concluded that the structural defects in off-stoichiometric TiC in the Mo–Ti–C ternary system result from the substitution of Mo at Ti sites and the formation of vacancies at C sites.

**Table 1:** Analyzed compositions and lattice parameters of the constituent phases in the alloys, together with the volume fraction of TiC in each alloy.

Bulk alloy composition (at%)			Tie line	Contituent phase	Composition (at%)			Lattice parameter (Å)	Volume fraction of the TiC phase
Mo	Ti	C			Mo	Ti	C		
Bal	4.8	0.6	Tie line 1	Mo	94.7 ± 0.3	4.6 ± 0.4	0.6 ± 0.3	a = 3.148	0.00
Bal	15.0	15.0	Tie line 1	Mo	94.5 ± 0.7	4.8 ± 0.5	0.6 ± 0.3	a = 3.150	0.39
				TiC	19.1 ± 1.1	36.3 ± 0.5	44.6 ± 0.9	a = 4.303	
Bal	20.0	20.0	Tie line 1	Mo	93.3 ± 0.5	5.9 ± 0.1	0.8 ± 0.4	a = 3.147	0.50
				TiC	17.0 ± 0.3	38.5 ± 0.6	44.5 ± 0.9	a = 4.308	
Bal	12.6	0.9	Tie line 2	Mo	86.9 ± 0.5	12.0 ± 0.4	1.1 ± 0.7	a = 3.149	0.00
Bal	23.0	15.0	Tie line 2	Mo	87.2 ± 0.7	12.1 ± 0.5	0.8 ± 0.3	a = 3.148	0.38
				TiC	9.9 ± 0.1	45.3 ± 0.1	44.8 ± 0.2	a = 4.316	
Bal	27.0	20.0	Tie line 2	Mo	88.6 ± 0.4	12.4 ± 0.3	1.0 ± 0.5	a = 3.149	0.51
				TiC	9.7 ± 0.0	45.2 ± 0.1	45.1 ± 0.1	a = 4.317	
Bal	20.3	1.1	Tie line 3	Mo	76.0 ± 0.6	22.6 ± 0.6	1.4 ± 0.6	a = 3.155	0.00
Bal	30.0	15.0	Tie line 3	Mo	79.1 ± 0.8	19.9 ± 0.9	1.0 ± 0.2	a = 3.155	0.39
				TiC	6.2 ± 0.3	50.2 ± 0.8	43.6 ± 1.0	a = 4.320	
Bal	37.9	25.0	Tie line 3	Mo	78.1 ± 0.7	20.8 ± 0.4	1.1 ± 0.3	a = 3.149	0.63
				TiC	5.8 ± 0.2	50.1 ± 0.6	44.1 ± 0.9	a = 4.318	
Bal	45.6	0.8	Tie line 4	Mo	52.3 ± 2.8	46.7 ± 2.7	1.0 ± 0.5	a = 3.172	0.00
Bal	50.0	15.0	Tie line 4	Mo	53.6 ± 0.2	45.6 ± 0.6	0.8 ± 0.4	a = 3.174	0.41
				TiC	2.3 ± 0.1	57.7 ± 0.6	40.0 ± 0.6	a = 4.322	
Bal	53.2	25.0	Tie line 4	Mo	53.3 ± 0.4	46.1 ± 0.3	0.7 ± 0.1	a = 3.175	0.67
				TiC	2.2 ± 0.4	57.7 ± 1.1	40.1 ± 0.7	a = 4.312	
–	Bal	5.0	Tie line 5	Ti	–	99.0 ± 0.1	1.0 ± 0.1	a = 2.951, c = 4.689	0.12
				TiC	–	64.0 ± 0.5	36.0 ± 0.8	a = 4.302	
–	Bal	10.0	Tie line 5	Ti	–	98.7 ± 0.1	1.3 ± 0.1	a = 2.954, c = 4.689	0.28
				TiC	–	64.5 ± 0.5	35.5 ± 0.9	a = 4.303	
–	Bal	15.0	Tie line 5	Ti	–	…	…	a = 2.953, c = 4.692	0.43
				TiC	–	…	…	a = 4.306

**Figure 4:**
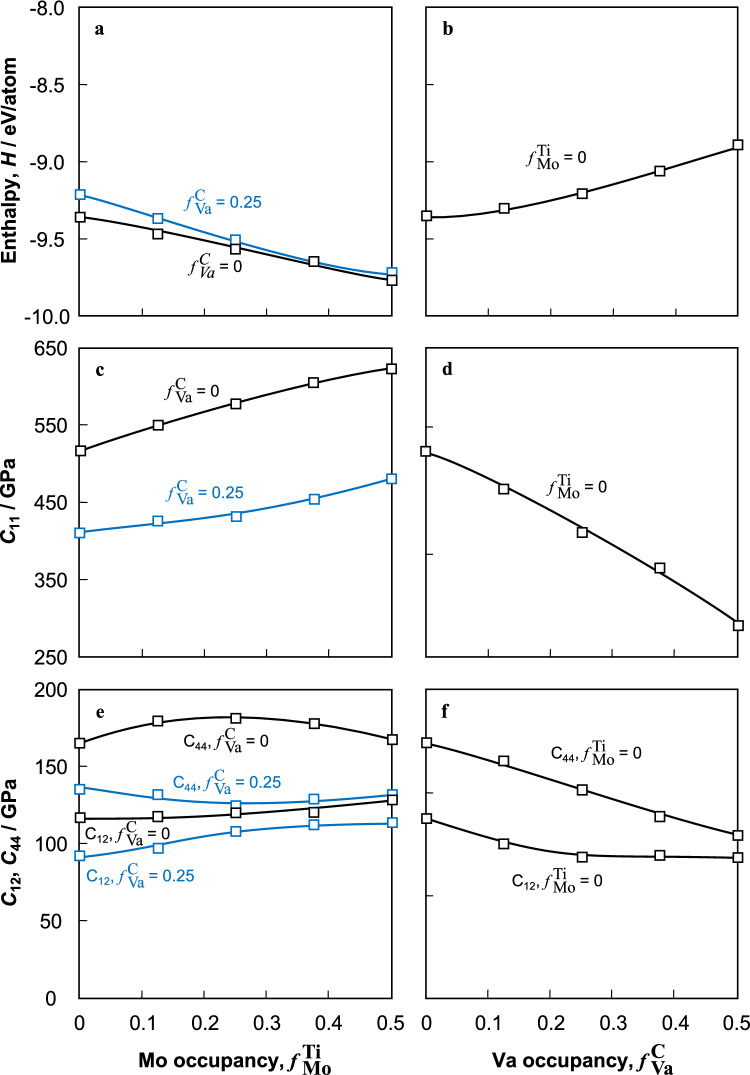
Changes in enthalpy and elastic constants calculated by DFT for (**a**,**c**,**e**) TiC with $f{}_{\text{Va}}^{ \, {\text{C}}}$ = 0, 0.25 as a function of $f{}_{\text{Mo}}^{ \, {\text{Ti}}}$ and (**b**,**d**,**f**) TiC with $f{}_{\text{Mo}}^{ \, {\text{Ti}}}$ = 0 as a function of $f{}_{\text{Va}}^{ \, {\text{C}}}$: (**a**,**b**) enthalpy, *H*, (**c**,**d**) *C*_11_, (**e**,**f**) *C*_12_ and *C*_44_.

Figure 4 shows the change in enthalpy (*H*) and the elastic constants obtained from DFT calculations for TiC as a function of $f{}_{\text{Mo}}^{ \, {\text{Ti}}}$ and $f{}_{\text{Va}}^{ \, {\text{C}}}$. *H* for TiC at $f{}_{\text{Va}}^{ \, {\text{C}}}$ = 0 and 0.25 decreased with increasing $f{}_{\text{Mo}}^{ \, {\text{Ti}}}$, whereas *H* for TiC with $f{}_{\text{Mo}}^{ \, {\text{Ti}}}$ = 0 increased with increasing $f{}_{\text{Va}}^{ \, {\text{C}}}$. The elastic constants of TiC basically increase and decrease as *H* decreases and increases, respectively. This is because *H* roughly corresponds to the cohesive energy as shown in the case of *C*_11_. On the other hand, *C*_44_ of TiC with $f{}_{\text{Va}}^{ \, {\text{C}}}$ = 0 and 0.25 did not always increase and, in some cases, decreased with decreasing *H*. As a result, especially for TiC with $f{}_{\text{Va}}^{ \, {\text{C}}}$ = 0.25, the difference between *C*_44_ and *C*_12_ became smaller as $f{}_{\text{Mo}}^{\text{ Ti}}$ increased. Similarly, the difference between *C*_44_ and *C*_12_ of TiC with $f{}_{\text{Mo}}^{ \, {\text{Ti}}}$ = 0 became smaller as $f{}_{\text{Va}}^{ \, {\text{C}}}$ increased. This is because the rate of decrease of *C*_12_ with increasing $f{}_{\text{Va}}^{ \, {\text{C}}}$ was more gradual than that of *C*_44_. *C*_44_ of TiC with $f{}_{\text{Mo}}^{ \, {\text{Ti}}}$ = 0 decreased with increasing $f{}_{\text{Va}}^{ \, {\text{C}}}$.

**Figure 5:**
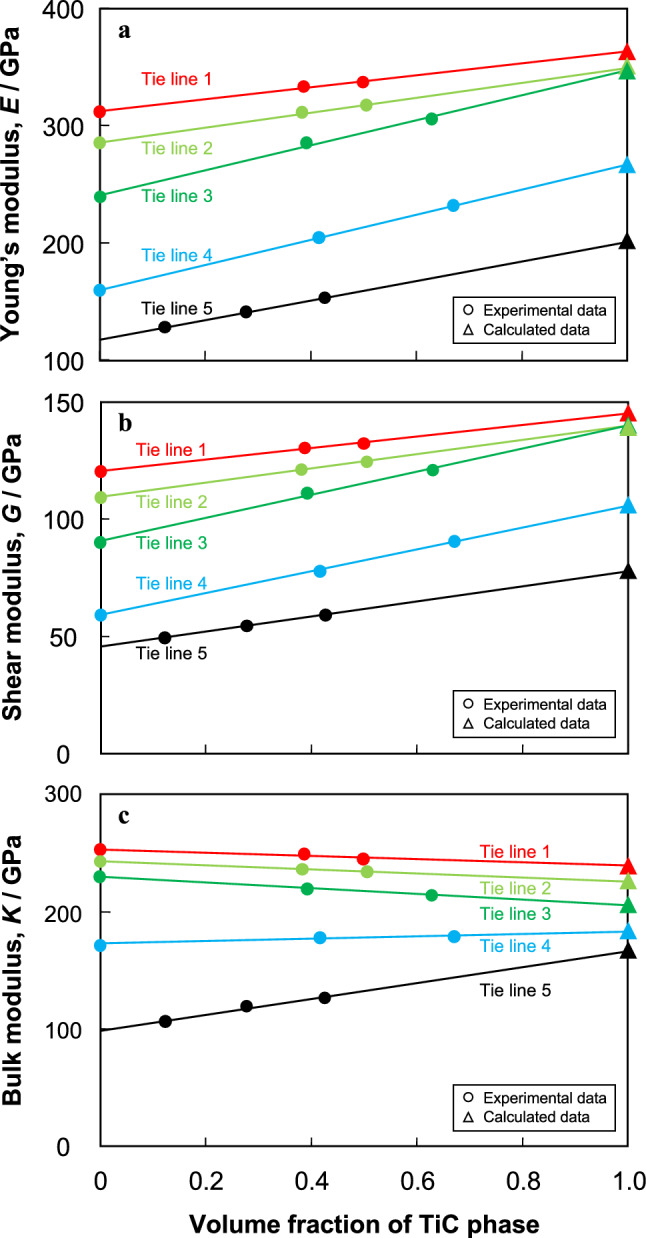
Changes in elastic moduli of alloys on Tie Lines 1–5 with the volume fraction of TiC: (**a**) Young’s modulus, *E*, (**b**) shear modulus, *G* and (**c**) bulk modulus, *K*. The elastic moduli change with the volume fraction of TiC; the elastic moduli of TiC with the terminal compositions described by the triangle symbols were calculated by using the rule of mixtures of the Voigt’s model.

The changes in *E*, *G*, and *K* experimentally obtained for bulk alloys on Tie Lines 1–5 are plotted as functions of the volume fraction of TiC (*V*_TiC_) in Fig. 5. The results are summarized in Table 2. All elastic moduli of the solid-solution phase (*V*_TiC_ = 0%) increased with increasing Mo content. The elastic moduli changed linearly with increasing *V*_TiC_. This means that the rule of mixtures of the Voigt model is applicable:

3 $$X{}_{\text{bulk}}\text{ = }X{}_{1}{V}_{1}+ \text{ } X{}_{2}{V}_{2},$$

where *X*_bulk_ is the elastic modulus of the bulk alloy, *X*_1_ and *X*_2_ are the elastic moduli of the constituent phases, and *V*_1_, *V*_2_ are the volume fractions of the constituent phases^59^. By using the rule of mixture, the elastic moduli of TiC with the terminal composition of each tie line can be estimated. The estimated elastic moduli and *K*/*G* of TiC on Tie Lines 1–5 are summarized in Table 3, along with $f{}_{{\text{M}}{\text{o}}}^{ \, {\text{Ti}}}$, $f{}_{\text{Va}}^{ \, {\text{C}}}$. The values of $f{}_{\text{Mo}}^{ \, {\text{Ti}}}$ and $f{}_{\text{Va}}^{ \, {\text{C}}}$ were calculated by using the average value for each terminal composition of TiC.

**Table 2:** Experimentally measured elastic moduli of the bulk alloys.

Bulk alloy composition (at%)			Tie line	Measured elastic modulus of bulk alloys			
Mo	Ti	C		Young's modulus, *E* (GPa)	Shear modulus, *G* (GPa)	Bulk modulus, *K* (GPa)	Poisson's ratio
Bal	4.8	0.6	Tie line 1	312.1 ± 1.2	120.5 ± 0.5	254 ± 1	0.295 ± 0.01
Bal	15.0	15.0	Tie line 1	333.2 ± 1.1	130.5 ± 0.5	249 ± 1	0.277 ± 0.01
Bal	20.0	20.0	Tie line 1	336.8 ± 0.7	132.5 ± 0.9	246 ± 6	0.271 ± 0.06
Bal	12.6	0.9	Tie line 2	285.6 ± 0.6	109.5 ± 0.3	243 ± 1	0.304 ± 0.01
Bal	23.0	15.0	Tie line 2	310.8 ± 0.6	121.3 ± 0.1	237 ± 3	0.282 ± 0.02
Bal	27.0	20.0	Tie line 2	317.6 ± 0.4	124.7 ± 0.1	234 ± 2	0.274 ± 0.01
Bal	20.3	1.1	Tie line 3	239.3 ± 0.4	90.2 ± 0.4	230 ± 12	0.327 ± 0.09
Bal	30.0	15.0	Tie line 3	285.6 ± 4.8	111.2 ± 2.4	221 ± 3	0.284 ± 0.06
Bal	37.9	25.0	Tie line 3	305.6 ± 1.1	121.0 ± 0.2	215 ± 3	0.263 ± 0.02
Bal	45.6	0.8	Tie line 4	159.6 ± 0.8	59.3 ± 0.2	172 ± 3	0.345 ± 0.03
Bal	50.0	15.0	Tie line 4	204.3 ± 1.1	78.0 ± 0.5	179 ± 0	0.310 ± 0.02
Bal	53.2	25.0	Tie line 4	232.6 ± 0.5	90.6 ± 0.1	180 ± 3	0.284 ± 0.02
–	Bal	5.0	Tie line 5	128.5 ± 0.7	49.5 ± 0.5	107 ± 3	0.299 ± 0.05
–	Bal	10.0	Tie line 5	141.8 ± 4.0	54.4 ± 1.6	120 ± 2	0.303 ± 0.02
–	Bal	15.0	Tie line 5	153.8 ± 2.0	59.3 ± 1.0	127 ± 1	0.298 ± 0.04

**Table 3:** Terminal compositions, atomic ratios, and calculated elastic moduli of TiC on Tie Lines 1–4. The terminal composition is the average for the two alloys on each tie line.

Tie line	Mo fraction at Ti site	Structure defect fraction at C site	Calculated elastic modulus of the TiC phase			
			Young's modulus, *E* (GPa)	Shear modulus, *G* (GPa)	Bulk modulus, *K* (GPa)	Poisson's ratio
Tie line 1	0.33	0.20	363.3	145.2	239.2	0.25
Tie line 2	0.18	0.18	349.7	139.7	226.5	0.25
Tie line 3	0.11	0.22	347.5	140.2	205.6	0.24
Tie line 4	0.04	0.33	266.3	105.7	184.3	0.26
Tie line 5	0	0.44	201.8	77.6	166.8	0.30

**Figure 6:**
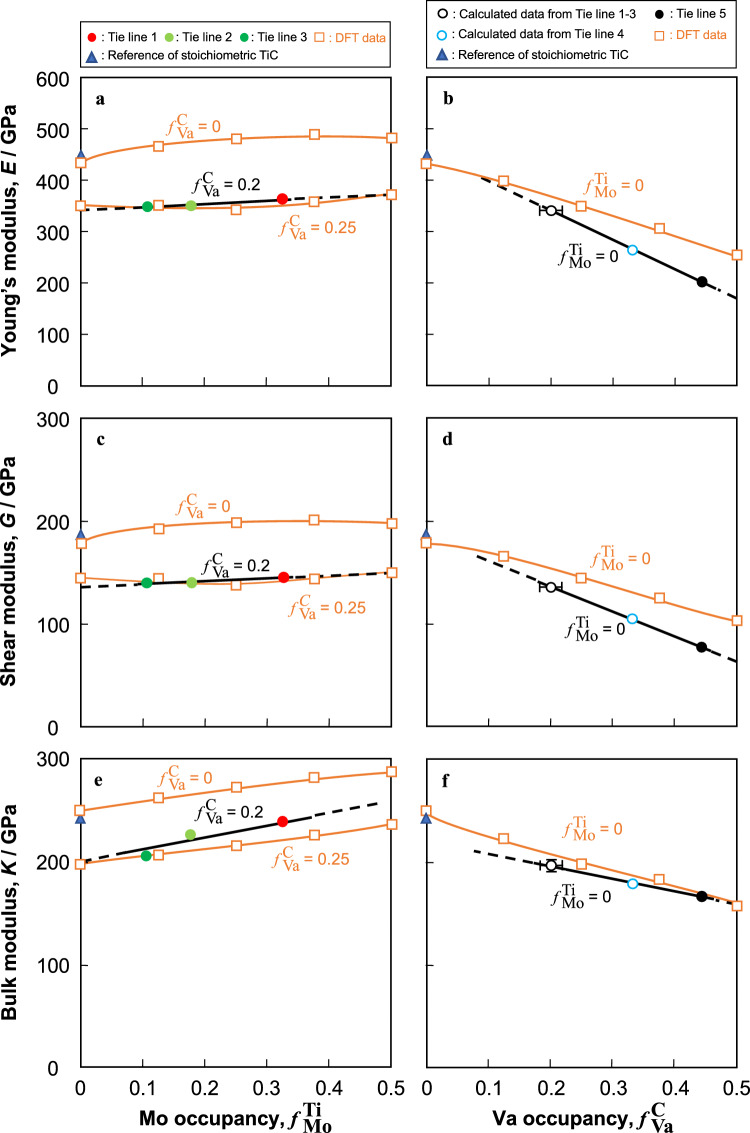
Changes in the elastic moduli of TiC obtained from experimental data and DFT calculations, together with reference data for stoichiometric TiC^59,60^: (**a**,**b**) Young’s modulus, *E* (**c**,**d**) shear modulus, *G*, (**e**,**f**) bulk modulus, *K*. (**a**,**c**,**e**) Changes in elastic moduli with $f{}_{\text{Mo}}^{\text{Ti}}$ in TiC with specific value of $f{}_{\text{Va}}^{ \, {\text{C}}}$. (**b**,**d**,**f**) Changes in elastic moduli with $f{}_{\text{Va}}^{ \, {\text{C}}}$ in TiC with specific vacancy value of $f{}_{\text{Mo}}^{\text{Ti}}$.

Details of the changes in elastic modulus with substitution by Mo at Ti sites and vacancy formation at C sites will now be discussed. Figure 6 shows the changes in *E*, *G* and *K* obtained from the experimental results and DFT calculations as functions of $f{}_{\text{Mo}}^{\text{Ti}}$ and $f{}_{\text{Va}}^{ \, {\text{C}}}$^60,61^. Since the values of $f{}_{\text{Va}}^{ \, {\text{C}}}$ for Tie Lines 1–3 are almost the same ($f{}_{\text{Va}}^{ \, {\text{C}}}$ = 0.2), the elastic moduli of TiC on Tie Line 1–3 were dependent on the Mo fraction (Fig. 6a,c,e). The vacancy fraction dependence was determined from the elastic modulus of binary TiC (Tie Line 5), as well as that of ternary TiC, calculated as $f{}_{\text{Mo}}^{\text{Ti}}$ = 0 by using the Mo fraction dependence (Fig. 6b,d,f). The value of *K* in the experimental results at $f{}_{\text{Va}}^{ \, {\text{C}}}$ = 0.2 increased with increasing $f{}_{\text{Mo}}^{\text{Ti}}$, whereas* G* and *K* were almost constant, regardless of $f{}_{\text{Mo}}^{\text{Ti}}$ (Fig. 6a,c,e). *E*,* G*, and* K* of the experimental results at $f{}_{\text{Mo}}^{\text{Ti}}$ = 0 decreased with increasing $f{}_{\text{Va}}^{ \, {\text{C}}}$ and the degree of change in *E* and *G* was larger than that in* K* (Fig. 6b,d,f). Note that the experimental results and DFT calculations were in good agreement. Moreover, the reported DFT data for binary TiC^31,35,39^ and (Ti,Mo)C^41^ also show similar tendencies. Therefore, *E* and *G* are highly dependent on the fraction of vacancies, whereas *K* is highly dependent on the fraction of Mo.

## Discussion

**Figure 7:**
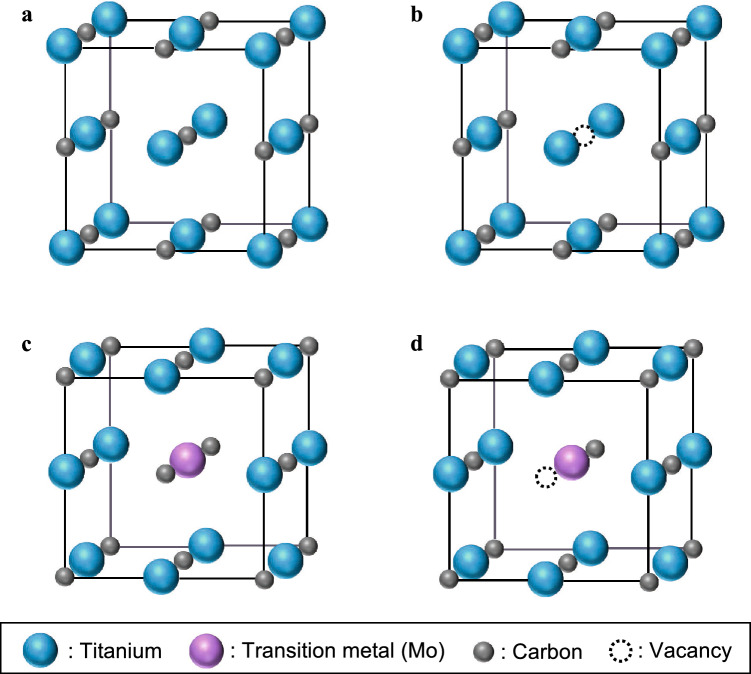
Structure of TiC phase: (**a**) stoichiometric TiC, (**b**) off-stoichiometric TiC that forms a vacancy at a C site, (**c**) off-stoichiometric TiC in which Mo is substituted at a Ti site, (**d**) off-stoichiometric TiC that forms a vacancy at a C site and substitutes a Mo at a Ti site.

Here, the factors controlling the elastic moduli of TiC are discussed in relation to the bond strength. Figure 7 shows the structures of TiC with and without off-stoichiometry. In stoichiometric TiC, Ti atoms at the Ti sites and C atoms at the C sites form six nearest neighbor (NN) Ti–C (M–X) bonds, and twelve next nearest neighbor (NNN) Ti–Ti (M–M) and C–C (X–X) bonds (Fig. 7a).

When a vacancy is formed on a C site by off-stoichiometry, the six NN Ti–C bonds disappear (Fig. 7b), and the total NN bond strength decreases. Therefore, it is ready to understand that the elastic moduli decrease as $f{}_{\text{Va}}^{ \, {\text{C}}}$ increases. This tendency is also observed in MC_*x*_ carbides and MN_*x*_ nitrides^34,62,63^.

When a Mo atom substitutes into a Ti site in stoichiometric TiC, the six NN Ti–C bonds change to the NN Mo–C bonds and the NN bond strength should be changed (Fig. 7c). Here, it was observed that *G* and *E* are almost constant, but *K* increases with the substitution of Mo (Fig. 6a,c,e). These results suggest that not only NN bonds but also NNN bonds affect the elastic moduli and the degree of influence of NNN bonds on each elastic modulus is different. When a Mo atom substitutes at the Ti site in stoichiometric TiC, the twelve NNN Ti–Ti bonds change to twelve NNN Ti–Mo bonds. Furthermore, when a vacancy is formed at a C site neighboring the Mo atom, six NN M–C bonds and twelve NNN C–C bonds disappear (Fig. 7d) and the effect of the M–M bonds may become more significant. Here, it is inferred that the strength of the NNN Mo–Ti and Mo–Mo bonds are stronger than that of the Ti–Ti bond because the elastic modulus of the Mo phase decreases with increasing Ti content (Fig. 5). On the other hand, the Mo–C bond strength appears to be weaker than that of the Ti–C bond because *G* and *E* remain constant as the Mo fraction increases. On the other hand, *K* increases with increasing Mo fraction even if vacancies are formed (Fig. 6a,c,e). This is because the effect of M–M bond strength on* K* is greater than that of *G* and *E*. Therefore, *K* can be increased by increasing the NNN M–M bond strength. A similar phenomenon was observed in (Ti, W)C. The values of *E* and *G* for TiC and (Ti_0.5_, W_0.5_)C are almost identical, whereas *K* for (Ti_0.5_, W_0.5_)C is significantly higher (312 GPa)^41^ than that for TiC or (Ti_0.5_, Mo_0.5_)C, as calculated in this study. This can be reasonably explained by the fact that the elastic modulus of W is higher than that of Ti and Mo, which increases the NNN M–M bond strength^64^. Furthermore, this idea rationalizes the facts that VC and TaC have almost the same values of *E* and *G***,** whereas the value of *K* for TaC is higher than that for VC: this is because the elastic modulus of Ta is higher than that of V^35,64^. Further investigations of the strength of M–M and M–X bonds are needed to clarify the factors controlling the elastic properties of B1-type MX compounds.

The change in the elastic moduli of the B1-type MX, TiC, with off-stoichiometry was investigated experimentally and by DFT calculations for Mo–Ti–C ternary alloys. Our conclusions can be summarized as follows.

1. The elastic moduli of off-stoichiometric TiC in equilibrium with a solid-solution phase can be measured experimentally from the rule of mixtures of the Voigt model.
2. The elastic moduli of off-stoichiometric TiC at room temperature can be predicted by DFT calculations.
3. The bulk modulus (*K*) of TiC increases with increasing Mo fraction at Ti sites, whereas the Young’s modulus (*E*) and shear modulus (*G*) remain almost constant. On the other hand, all the elastic moduli decrease with increasing the fraction of vacancies at C sites. These results suggest that the M–X bond strength should be the dominant factor in these moduli and the effect of M–M bond on* K* is greater than that of *G* and *E*.

## Methods

### Experimental procedure

The compositions of the alloys studied, expressed as atomic percentages, were Mo–(4.8–53.2)%Ti–(0.6–25.0)%C in the (Mo,Ti)/TiC two-phase region or the Mo–Ti single-phase region in the Mo–Ti–C ternary system, and Ti–(5, 10, 15)% C of the Ti/TiC two-phase region in the Ti–C binary system^19,56,57^. (Hereafter, all compositions are expressed as atomic percentages). These alloys were prepared as 9–10 cm^3^ ingots from pure Mo (99.99 wt%), Ti (99.9 wt%), and TiC (99 wt%) by conventional arc melting under an Ar atmosphere. Each ingot was melted five times and turned over each time to prevent segregation. To ensure that phase equilibria were attained, heat treatment in an Ar atmosphere was performed at 1800 °C for 72 h for the Mo–Ti–C ternary alloys and at 1500 °C for 72 h for the Ti–C binary alloys; this was followed by furnace cooling. The microstructure of the alloys was examined by scanning electron microscopy (SEM) and transmission electron microscopy (TEM). TEM disks with a thickness of 0.1 mm and a diameter of 3 mm were machined and mechanically polished. These were then subjected to dimple grinding followed by ion milling. Phase identification and lattice-parameter measurements of the constituent phases were conducted by X-ray diffractometry. Compositional analyses of the phases were performed by using a field-emission electron-probe microanalyzer (EPMA) equipped with a wavelength-dispersive X-ray spectroscope (WDX) at 10 kV and 5.0 × 10^−8^ Å. Details of the compositional analyses are described elsewhere^19^. The elastic parameters of the alloys after heat treatment were measured by the electromagnetic acoustic resonance (EMAR) method, assuming an isotropic elastic medium. The elastic moduli measurements were performed at room temperature in a magnetic field of 0.5 T in a frequency range of 200–1400 kHz with a step frequency of 1 kHz. Details of the EMAR measurements and analysis are also presented elsewhere^65^.

### Computational details

The Vienna ab initio simulation package (VASP)^66^ was used to perform the DFT calculations within the generalized gradient approximation of Perdew, Burke, and Ernzerhof (GGA-PBE)^67^. Electron–ion interactions were modeled by using the projector-augmented wave potentials^68^, and the total energies were minimized and converged to within 10^–5^ eV/atom. The *k*-point grids for the Monkhorst–Pack method^69^ and the cut-off energy were set to 6 × 6 × 6 and 600 eV, respectively.

The formation energies of B1-type Mo_*x*_Ti_1–*x*_C, TiC_1–*y*_, and Mo_*x*_Ti_1−*x*_C_0.75_ were calculated across the full composition range (0 < *x*, *y* < 1) by using the following equation:

4 $$\Delta H = E_{{{\text{Mo}}_{x} {\text{Ti}}_{{1{-}x}} {\text{C}}_{{1{-}y}} { }}} - xE_{{{\text{Mo}}}} - (1 - x)E_{{{\text{Ti}}}} - (1 - y)E_{{\text{C}}} ,$$

where *x* is the fraction of Mo at Ti sites, *y* is the fraction of vacancies of the C sites, and *E*_X_ is the total energy of X per atom. The special quasi-random structure was obtained by using the *Alloy Theoretic Automated Toolkit* (ATAT)^70^: The number of atoms *n* in the supercells was 64 with Mo concentrations *x* = 0.00, 0.125, 0.250, 0.375, 0.500, and 1.000, and vacancy concentrations *y* = 0.00, 0.125, 0.250, 0.375, 0.500, and 1.000.

The elastic constants *C*_11_, *C*_12_, and *C*_44_ were obtained by fitting the calculated strain energy–strain curves with strain (*δ*) *δ* = ± 0.001 and ± 0.002. The various elastic constants were calculated by using the following equations:

5 $${\overline{C{}}}_{11}\text{ = }\frac{C{\text{}}_{11}\text{ + }C{\text{}}_{22}\text{ + }C{\text{}}_{33}}{3},$$

6 $${\overline{C\text{}}}_{12}\text{ = }\frac{C{\text{}}_{12}\text{ + }C{\text{}}_{13}\text{ + }C{\text{}}_{23}}{3},$$

7 $${\overline{C\text{}}}_{44}\text{=}\frac{C{\text{}}_{44}\text{ + }C{\text{}}_{55}\text{ + }C{\text{}}_{66}}{3}.$$

The isotropic Young’s modulus (*E*), bulk moduli (*K*) and shear moduli (*G*) were determined based on the Voigt–Reuss–Hill approach^71^ and calculated from the elastic constants given above and the following equations:

8 $$E = \frac{{(\overline{C}_{11} {-} \overline{C}_{12} ) (\overline{C}_{11} + 2\overline{C}_{12} )}}{{(\overline{C}_{11} + \overline{C}_{12} )}},$$

9 $$G{}\text{ = }\frac{1}{ \, {\overline{C\text{}}}_{44}},$$

10 $$K = \frac{GE}{{3 (3G {-} E)}}.$$

## References

[1] Storms EK (1967). The Refractory Carbides.

[2] Rajabi A, Ghazali MJ, Daud AR (2015). Chemical composition, microstructure and sintering temperature modifications on mechanical properties of TiC-based cermet: A review. Mater. Des..

[3] Dubrovinskaia NA, Dubrovinsky LS, Saxena SK, Ahuja R, Johansson B (1999). High-pressure study of titanium carbide. J. Alloys Compd..

[4] Garcia J, Pitonak R (2013). The role of cemented carbide functionally graded outer-layers on the wear performance of coated cutting tools. Int. J. Refract. Met. Hard Mater..

[5] Veprek S, Veprek-Heijman MGJ, Karvankova P, Prochazka J (2005). Different approaches to superhard coatings and nanocomposites. Thin Solid Films.

[6] Durlu N (1999). Titanium carbide based composites for high temperature applications. J. Eur. Ceram. Soc..

[7] Le Flem M, Allemand A, Urvoy S, Cédat D, Rey C (2008). Microstructure and thermal conductivity of Mo–TiC cermets processed by hot isostatic pressing. J. Nucl. Mater..

[8] Compton BG, Zok FW (2013). Impact resistance of TiC-based cermets. Int. J. Impact Eng..

[9] Endo H, Ueki M, Kubo H (1991). Microstructure and mechanical properties of hot-pressed SiC–TiC composites. J. Mater. Sci..

[10] Maerky C, Guillou M-O, Henshall JL, Hooper RM (1996). Indentation hardness and fracture toughness in single crystal TiC_0.96_. J. Mater. Sci. Eng. A.

[11] Perepezko JH (2009). The hotter the engine, the better. Science.

[12] Pollock TM (2016). Alloy design for aircraft engines. Nat. Mater..

[13] Pedture NP (2016). Advanced structural ceramics in aerospace propulsion. Nat. Mater..

[14] Tang S, Hu C (2017). Design, preparation and properties of carbon fiber reinforced ultra-high temperature ceramic composites for aerospace applications: A review. J. Mater. Sci Technol. (Shenyang, China)..

[15] Fahrenholtz WG, Hilmas GE (2017). Ultra-high temperature ceramics: Materials for extreme environments. Scr. Mater..

[16] Marshall DB, Cox BN (2008). Integral textile ceramic structures. Annu. Rev. Mater. Res..

[17] Jin X, Fan X, Lu C, Wang T (2018). Advanced in oxidation and ablation resistance of high and ultra-high temperature ceramics modified or coated carbon/carbon composite. J. Eur. Ceram. Soc..

[18] Pietzka MA, Schuster JC (1994). Summary of constitutional data on the aluminum–carbon–titanium system. J. Phase Equilib..

[19] Ida S, Sekido N, Yoshimi K (2020). Solidification pathways and phase equilibria in the Mo–Ti–C ternary system. High Temp. Mater. Processes (Berlin Ger.)..

[20] Bradley AJ, Taylor A (1937). An X-ray analysis of the nickel–aluminum system. Proc. R. Soc. Lond. Ser. A.

[21] Westbrook JH (1956). Temperature dependence of hardness of the equi-atomic iron group aluminides. J. Electrochem. Soc..

[22] Westbook JH (1957). Defect structure and the temperature dependence of hardness of an intermetallic compound. J. Electrochem. Soc..

[23] Ball A, Smallman RE (1966). The deformation properties and electron microscopy studies of the intermetallic compound NiAl. Acta Metall..

[24] Yang WJ, Lin F, Dodd RA (1978). Structure of vacancy-defective NiAl. Scr. Metall..

[25] Tan Y, Shinoda T, Mishima Y, Suzuki T (1993). Defect hardening by the deviation from stoichiometry in NiAl. Nippon Kinzoku Gakkaishi.

[26] Pike LM, Chang YA, Liu CT (1997). Solid-solution hardening and softening by Fe additions to NiAl. Intermetallics.

[27] Hahn KH, Vedula K (1989). Room temperature tensile ductility in polycrystalline B2 NiAl. Scr. Metall..

[28] Sundgren J-E (1985). Structure and properties of TiN coatings. Thin Solid Films.

[29] Williams WS (1964). Scattering of electron by vacancy in nonstoichiometric crystals of titanium carbide. Phys. Rev. A.

[30] Williams WS (1971). Transition-metal carbides. Prog. Solid State Chem..

[31] Williams WS (1997). Transition metal carbides, nitrides, and borides for electronic applications. JOM.

[32] Lye RG, Logothetis EM (1966). Optical properties and band structure of titanium carbide. Phys. Rev..

[33] Ramqvist L, Hamrin K, Johansson G, Gelius U, Nordling C (1970). VC, NbC and TaC with varying carbon content studied by ESCA. J. Phys. Chem. Solids.

[34] Hugosson HW, Korzhavyi P, Jansson U, Johansson B, Eriksson O (2001). Phase stabilities and structural relaxations in stoichiometric TiC_1–__*x*_. Phys. Rev. B.

[35] Korzhavyi PA, Pourovskii LV, Hugosson HW, Ruban AV, Johansson B (2002). Ab initio study of phase equilibria in TiC_*x*_. Phys. Rev. Lett..

[36] Dridi Z, Bouhaf B, Ruterana P, Aourag H (2002). First-principles calculations of vacancy effects on structural and electronic properties of TiC_*x*_ and TiN_*x*_. J. Phys. Condens. Matter.

[37] Yu X-X, Thompson GB, Weinberger CR (2015). Influence of carbon vacancy formation on the elastic constants and hardening mechanisms in transition metal carbides. J. Eur. Ceram. Soc..

[38] Kindlund H, Sangiovanni DG, Lu J, Jensen J, Chirita V, Birch J, Petrov I, Greene JE, Hultman L (2014). Vacancy-induced toughening in hard single-crystal V_0.5_Mo_0.5_N_x_/MgO(001) thin films. Acta Mater..

[39] Razumovskiy VI, Ruban AV, Odqvist J, Dilner D, Korzhavyi PA (2014). Effect of carbon vacancies on thermodynamic properties of TiC–ZrC mixed carbides. CALPHAD Comput. Coupling Phase Diagr. Thermochem..

[40] Jhi S-H, Ihm J, Louie SG, Cohen ML (1999). Electronic mechanism of hardness enhancement in transition-metal carbonitrides. Nature.

[41] Edstöm D, Sangiovanni DG, Hultman L, Petrov I, Greene JE, Chirita V (2018). Elastic properties and plastic deformation of TiC- and VC-based pseudobinary alloys. Acta Mater..

[42] Li Y, Katsui H, Goto T (2019). Phase decomposition of (Ti, Zr)(C, N) solid solutions prepared by spark plasma sintering. J. Eur. Ceram. Soc..

[43] Cap Z, Jin N, Ye J, Du X, Liu Y (2020). First-principles study on the effects of N and Al doping on the mechanical properties and electronic structures of TiC. RSC Adv..

[44] Ida S, Watanabe K, Yoshimi K (2023). Solidification microstructure and mechanical properties of B1-type TiC in Fe–Ti–C ternary alloys. Tetsu to Hagane.

[45] Lu Y, Watanabe M, Miyata R, Nakamura J, Yamada L, Kato H, Yoshimi K (2020). Microstructures and mechanical properties of TiC-particulate-reinforced Ti–Mo–Al intermetallic matrix composites. Mater. Sci. Eng. A.

[46] Miyamoto S, Yoshimi K, Ha S, Kaneko T, Nakamura J, Sato T, Maruyama K, Tu R, Goto T (2014). Phase equilibria, microstructure, and high-temperature strength of TiC-added Mo–Si–B Alloys. Metall. Mater. Trans. A.

[47] Kamata SY, Kanekon D, Lu Y, Sekido N, Maruyama K, Eggeler G, Yoshimi K (2018). Ultrahigh-temperature tensile creep of TiC-reinforced Mo–Si–B-based alloy. Sci. Rep..

[48] Moriyama T, Yoshimi K, Zhao M, Masnou T, Yokoyama T, Nakamura J, Katsui H, Goto T (2017). Room-temperature fracture toughness of MoSiBTiC alloys. Intermetallics.

[49] Yamamoto S, Yoshimi K, Kim J, Yokoyama K (2016). Effects of microstructure on high-temperature strength of TiC-added Mo–Si–B alloys. Nippon Kinzoku Gakkaishi.

[50] Uemura S, Yamamuro T, Kim JW, Morizono Y, Tsurekawa S, Yoshimi K (2018). Quantitative evaluation of microstructure in Mo–Si–B–TiC alloy produced by melting and tilt casting method. Mater. Trans..

[51] Sangiovanni DG, Mellor W, Harrington T, Kaufmann K, Vecchio K (2021). Enhancing plasticity in high-entropy refractory ceramics via tailoring valence electron concentration. Mater. Des..

[52] Eibler R (2007). New aspects of the energetics of ordered Ti_2_C and Ti_2_N. J. Phys. Condens. Matter.

[53] Tsurekawa S, Yoshinaga H (1992). Identification of long range ordered Structure in TiC_0.59_ by transmission electron microscopy. Nippon Kinzoku Gakkaishi.

[54] Tsuda H, Ozaki T, Mori S (2020). Precipitation of titanium carbide particles dispersed in titanium matrix composites synthesized from Ti–C–N system powder mixtures using arc-melting method. Mater. Trans..

[55] Tsuda H, Ozaki T, Mori S (2012). Effects of chromium and nitrogen contents on microstructural changes in TiC particles in (α+β)- and β-titanium matrix composites. Mater. Trans..

[56] Bandyopadhyay D, Haldar B, Sharma RC, Chakraborti N (1999). The Ti–Mo–C (titanium–molybdenum–carbon) system. J. Phase Equilib..

[57] Pierson HO (1996). Handbook of Refractory Carbides and Nitrides: Properties, Processing and Applications.

[58] Teatum, E. T., Gschneidner, K. A., Waber, J. T. Compilation of calculated data useful in predicting metallurgical behavior of the elements in binary alloy systems, Los Alamos Science Laboratory, Los Alamos, NM (1968). https://www.osti.gov/servlets/purl/4789465 (accessed 1 Feb 2023).

[59] Voigt W (1928). Lehrbuch der Kristallphysik (mit Ausschluss der Kristalloptik).

[60] Chang R, Graham LJ (1966). Low-temperature elastic properties of ZrC and TiC. J. Appl. Phys..

[61] Yang Q, Lengauer W, Koch T, Scheerer M, Smid I (2000). Hardness and elastic properties of Ti(C_*x*_N_1–__*x*_), Zr(C_*x*_N_1–__*x*_) and Hf(C_*x*_N_1–__*x*_). J. Alloys Compd..

[62] Valeeva AA, Gusev AI (2021). Effect of nonstoichiometry on elastic properties of niobium carbide NbC_*y*_. J. Refract. Met. Hard Mater..

[63] Kral C, Lengauer W, Rafaja D, Ettmayer P (1998). Critical review on the elastic properties of transition metal carbides, nitrides and carbonitrides. J. Alloys Compd..

[64] Allard S (1969). International Tables of Selected Constants. Metals, Thermal and Mechanical Data.

[65] Zhao M, Yoshimi K, Maruyama K, Yubuta K (2014). Thermal vacancy behavior analysis through thermal expansion, lattice parameter and elastic modulus measurements of B2-type FeAl. Acta Mater..

[66] Kresse G, Hafner J (1993). Ab initio molecular dynamics for liquid metals. Phys. Rev. B.

[67] Perdew JP, Burke K, Ernzerhof M (1996). Generalized gradient approximation made simple. Phys. Rev. Lett..

[68] Blöchl PE (1994). Projector augmented-wave method. Phys. Rev. B.

[69] Monkhorst HJ, Pack JD (1976). Special points for Brillouin-zone integrations. Phys. Rev. B.

[70] van de Walle A, Tiwarya P, de Jong M, Olmsted DL, Asta M, Dick A, Shin D, Wang Y, Chen L-Q, Liu Z-K (2013). Efficient stochastic generation of special quasirandom structures. CALPHAD Comput. Coupling Phase Diagr. Thermochem..

[71] Hill R (1952). The elastic behaviour of a crystalline aggregate. Proc. Phys. Soc. Lond. Sect. A.

